# Peroneal tendons well vascularized: results from a cadaveric study

**DOI:** 10.1007/s00167-015-3946-4

**Published:** 2016-01-06

**Authors:** Pim A. D. van Dijk, F. Xavier Madirolas, Ana Carrera, Gino M. M. J. Kerkhoffs, Francisco Reina

**Affiliations:** Department of Orthopaedic Surgery, Orthopaedic Research Centre Amsterdam, Academical Medical Centre, University of Amsterdam, Amsterdam, The Netherlands; Academic Center for Evidence Based Sports Medicine, Amsterdam, The Netherlands; Amsterdam Collaboration on Health and Safety in Sports, Amsterdam, The Netherlands; Servicio de Cirugía Ortopédica y Traumatología, Hospital Universitario Josep Trueta, Girona, Spain; Medical Sciences Department, Clinical Anatomy, Embryology and Neuroscience Research Group (NEOMA), Faculty of Medicine, Girona University, Girona, Spain

**Keywords:** Tendon, Peroneal tendon, Tendon tear, Tendon rupture, Vincula, Vascularization, Blood supply

## Abstract

**Purpose:**

Peroneal tendon tears are relatively common injuries that seem to have a poor healing tendency. The discussion goes that peroneal tendons have avascular zones, contributing to the poor healing of those tears. The purpose of this study was to provide evidence on the vascularization pattern of the peroneal tendons.

**Methods:**

Ten adult fresh-frozen cadavers were obtained from a university-affiliated body donation programme. The femoral artery was injected with natural coloured latex at the level of the knee. Macroscopic and microscopic dissections were performed to visualize the vascularization towards the peroneal tendons. To expose intratendinous vascularity, the tendons were cleared using a modified Spälteholz technique.

**Results:**

In all specimens, blood was mainly supplied by the peroneal artery through a posterolateral vincula connecting both tendons. Branches were bifurcated every 3.9 ± 1.8 cm, starting 24 ± 5.3 cm proximal to the tip of the fibula. Eight out of 10 (80 %) specimens had poor vascularized zones in the peroneus longus tendon. No avascular zones were found in the peroneus brevis tendon.

**Conclusion:**

The peroneal tendons are well vascularized by the peroneal artery, via vessels running through a common vincula for both tendons. In the peroneus brevis, no avascular zones were found. To keep the tendons well vascularized and therefore improve tendon healing, surgeons should be careful leaving the vincula intact during surgical procedures.

## Introduction

Peroneal tendon tears are relatively common disorders that seem to have a poor healing tendency. It has been discussed in the literature that the peroneal tendons have avascular zones at the level of the most common locations for tears, contributing to the pathogenesis and poor healing of those tears [16, 19, 21, 27]. However, there is controversy regarding the existence of avascular regions. In order to further understand different peroneal tendon pathologies, the first step is to understand the peroneal tendons’ vascularization pattern. Pathophysiology of peroneal tendon tears can be acute or chronic in nature [7, 16, 28]. While acute tears are mostly attributed to sports injuries and lateral ankle instability [14], chronic tears are more likely a result of impingement, chronic subluxation or stenosis [3, 11, 14, 15]. Tendon degeneration is proposed as an underlying mechanism of injury [4, 15, 22, 29]. Reduced blood supply seems to play an important role in tendon degeneration. Therefore, understanding of the blood supply of the peroneal tendons is essential to understand the pathway of pathophysiology, healing and to optimize surgical treatment.

The literature attributes the vascularization of the peroneal tendons to different branches of the peroneal artery and the anterior tibial artery. Contribution of vessels from either the lateral tarsal artery [6, 20] or branches of the medial tarsal artery [26] remains controversial in literature. The blood vessels penetrate the tendons via one or two vincula from the posterolateral side to facilitate intratendinous blood supply [19, 26, 31]. According to van Dijk and Kort [31], the distal fibers of the peroneus brevis (PB) muscle belly transform to these vincula, ending approximately at the tip of the fibula.

Petersen et al. [19] proposed that the peroneal tendons have three critical avascular zones. One avascular zone was found in the region where the peroneus brevis tendon curls around the lateral malleolus. In the peroneus longus (PL) tendon, two avascular zones were found: one where the tendon curls around the lateral malleolus and the other where the tendon turns around the cuboid. These zones are consistent with the locations where peroneal tendon tears occur most frequent and healing tendency is poor [19]. In contrast, Sobel et al. [26] found no proof for avascular zones within the tendons. Both aforementioned studies only contained few specimens, and the accuracy of injection techniques varied. Hence, the vascularization pattern of the peroneal tendons remains a subject of controversy and discussion [14].

To create better insight in the blood supply of the peroneal tendons and thus to create a better understanding of the pathophysiology, healing and to optimize treatment, the purpose of the current study is to analyse the arterial anatomy of the peroneal tendons in cadavers. The hypothesis is that the peroneal tendons are well vascularized and free of avascular zones.

## Materials and methods

Ten adult fresh-frozen cadaveric lower extremities were obtained from a university-affiliated body donation program following the legal procedures and ethical framework governing the body donation in Spain. All specimens were free of scars at the lateral side of the ankle, and tendons were free of macroscopically visible tears, ruptures or degenerative changes. Since all donations were anonymous, no information was available on gender or prior pathologies. The age of all specimens ranged between 65 and 78 years. Before starting the intravascular injection and dissection procedure, legs were thawed to room temperature.

### Intravascular injection

The femoral artery was injected with natural coloured latex at the level of the knee by a cannula. Injection was performed under pulsatile manual pressure similar to the arterial blood pressure. To promote perfusion trough the smallest blood vessels, the lower legs were massages thoroughly. Small incisions were made in the tip of the toes to check whether the latex penetrated the smallest vessels.

### Dissection

Dissection was done at the posterolateral side of the lower limb using the fifth metatarsal, fibular groove and the fibular head as reference. First, the skin, the subcutaneous fat tissue and the fascia were removed to expose the arteries contributing to the vascularization of the peroneal tendons.

Microdissection was completed using a surgical microscope to expose the smaller vessels (Kaps SOM 62, Germany). The tendon sheaths were opened in a longitudinal direction and the vincula was carefully studied. Dissection was completed in a structured manner, with photographs taken during the dissection. All measurements were taken using a digital caliper (Digimatic Caliper, Mitutoyo, Japan; 0.01 accuracy). Three observers individually obtained the measurements to minimize intraobserver error. All measurements were rounded to millimeters.

### Spälteholz technique

To visualize intratendinous vascularization, four specimens were prepared using the Spälteholz technique, which provides transparent three-dimensional structures. The peroneal muscles and tendons were isolated together with the peroneal artery and its branches after dissection and excised together with the fibula as an anatomical reference. After complete dehydration, benzyl benzoate and methyl salicylate were used to clear the specimens satisfactorily and to reveal the microvascularization of the peroneal tendons. The tendons were studied with a surgical microscope by three independent observers, after which the tendons were photographed and measurements were taken carefully.

### Statistical analysis

Descriptive statistics were used in calculating means and standard deviations for distances between the origins of branches. One-way analysis of variance was used to compare group means in distance. A Bonferroni test was used when findings with the ANOVA model were significant. A *p* - value of less than 0.005 (0.05 divided by 10) was considered as statistically significant. Statistical analysis was performed using Stata version 13.0 software (STATA Corp., TX, USA).

## Results

**Fig. 1:**
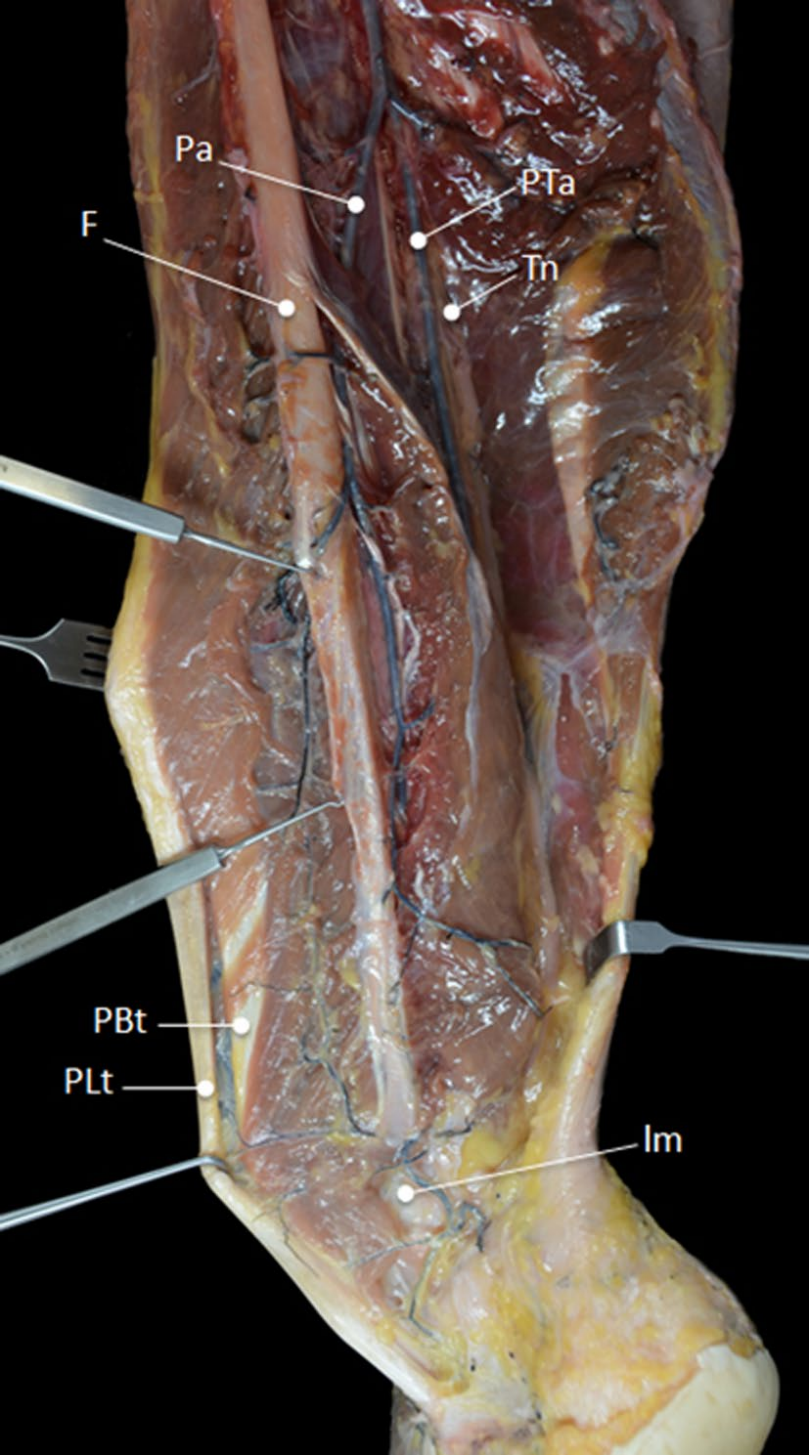
Main vascularization of the peroneal tendons is supplied by the peroneal artery. *Pa* peroneal artery, *PTa* posterior tibial artery, *F* fibula, *Tn* tibial nerve, *PBt* peroneus brevis tendon, *PLt* peroneus longus tendon, *lm* lateral malleolus

**Fig. 2:**
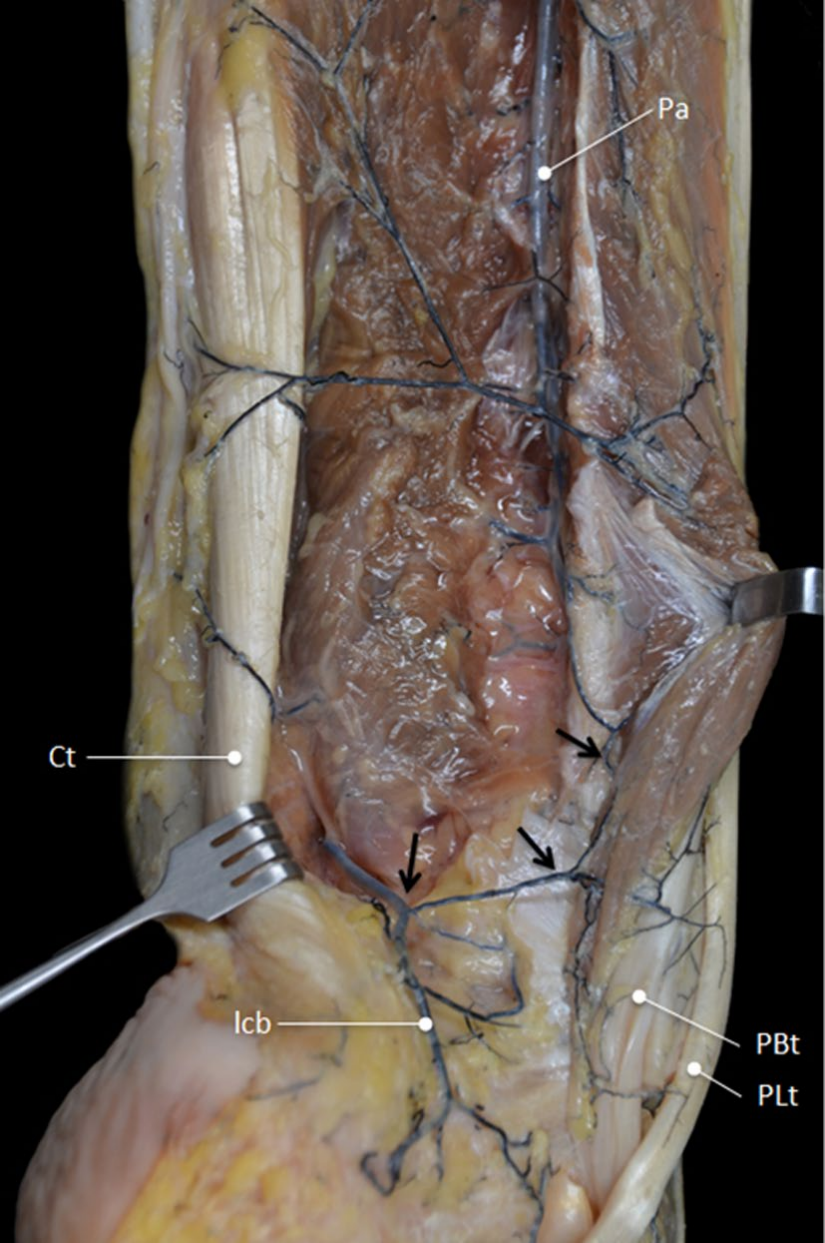
In 6 cases, a communicating branch was found between the peroneal artery and the posterior tibial artery (*arrows*). *Pa* peroneal artery, *Ct* calcaneal tendon, *lcb* lateral calcaneal branch, *PBt* peroneus brevis tendon, *PLt* peroneus longus tendon

**Fig. 3:**
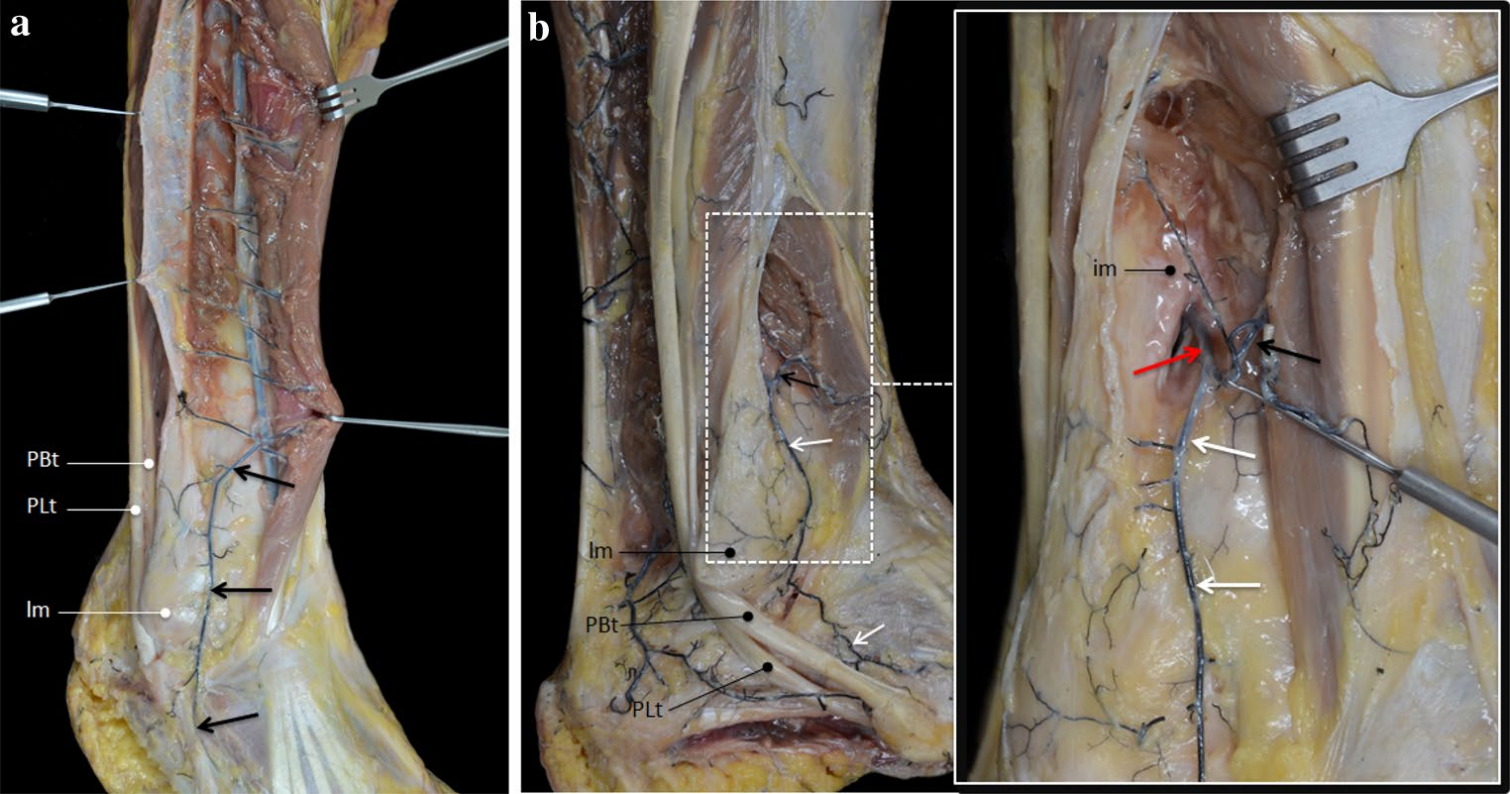
**a** In  6 cases, the main vascularization of the PL tendon at the dorsolateral area of the foot was supplied by the malleolar branch of the anterior tibial artery (*arrows*). *PBt* peroneus brevis tendon, *PLt* peroneus longus tendon, *lm* lateral malleolus. **b** In 4 cases, the PL was vascularized by vessels from the perforating branch of the peroneal artery (*red arrow*) that crosses the interosseous membrane and anastomoses with the malleolar branch of the anterior tibial artery (*black arrow*). *lm* lateral malleolus, *PBt* peroneus brevis tendon, *PLt* peroneus longus tendon, *im* interosseous membrane

**Fig. 4:**
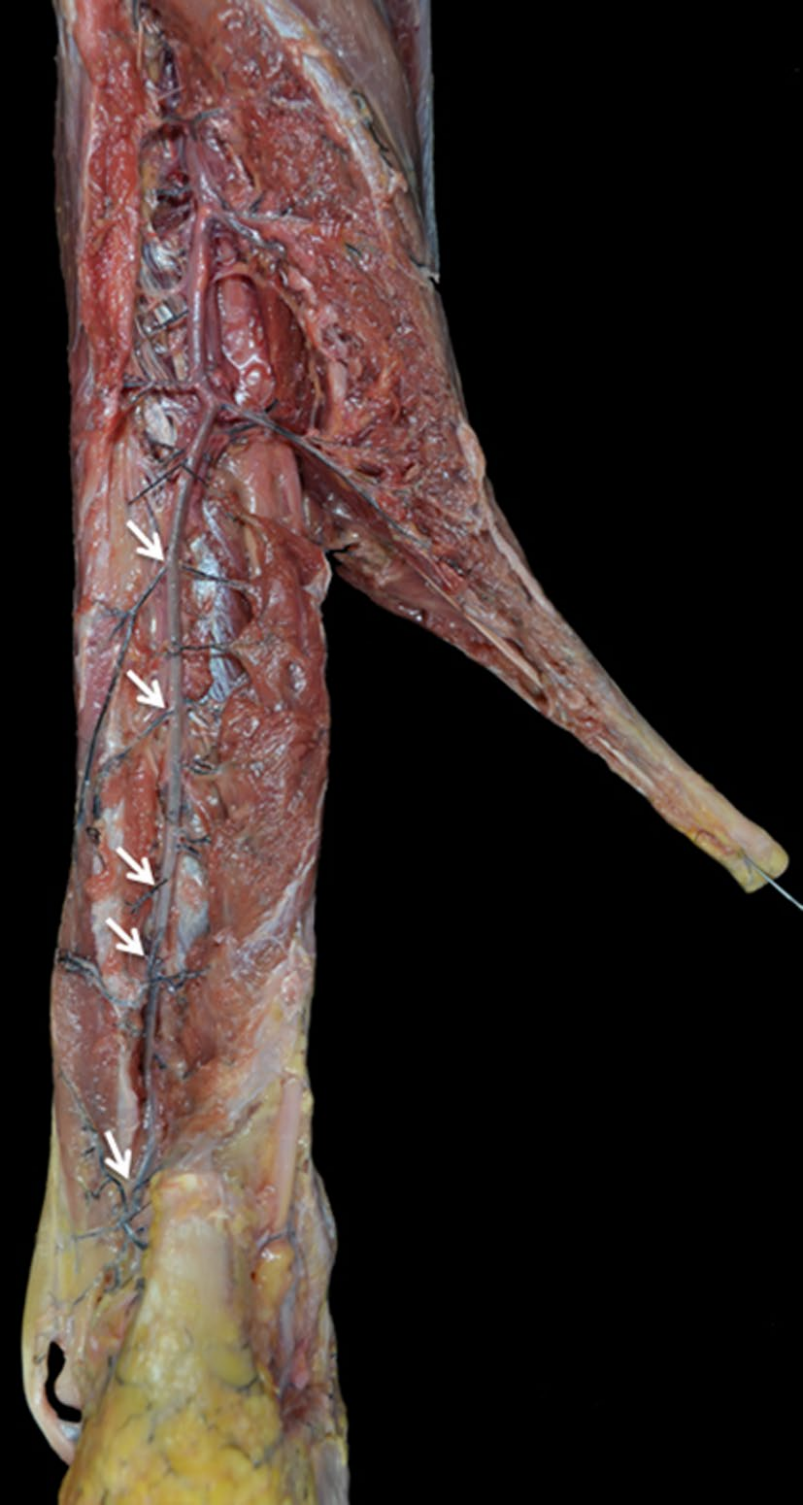
Peroneal artery splits of different branches (*arrows*) to enter the peroneal tendons

In all specimens, both tendons were mainly vascularized by the peroneal artery (Fig. 1). In 6 cases, a communicating branch was found between the peroneal artery and the posterior tibial artery before entering the tendons (Fig. 2). The anterior lateral malleolar branch of the anterior tibial artery was the main vessel supplying blood to the PL tendon at the dorsolateral area of foot (Fig. 3a). In 4 cases, vascularization of the PL tendon within the dorsolateral area was contributed by vessels from the perforating branch of the peroneal artery that crossed the interosseous membrane and anastomosed with the anterior lateral malleolar branch of the anterior tibial artery (Fig. 3b). Distances between the branches from the peroneal artery were measured (Fig. 4). The mean distance between the most proximal branch and the fibular tip was 24 ± 5.3 cm. The mean distance between the different branches was 3.9 ± 1.8 cm (Table 1). There was no difference between distances of branches.

**Table 1:** Distances between the different branches splitting off the peroneal artery

Distance	Mean ± SD (cm)	Distance with distal branch (cm)
Fibular tip–arch	3.7 ± 2.0	–
Fibular tip–first branch	8.1 ± 2.6	4.2 ± 2.1
Fibular tip–second branch	13 − 4.0	4.9 ± 2.7
Fibular tip–third branch	17 ± 3.9	3.8 ± 0.94
Fibular tip–fourth branch	21 ± 5.3	3.7 ± 1.8
Fibular tip–fifth branch	24 ± 5.3	3.2 ± 1.1

*SD* Standard Deviation

**Fig. 5:**
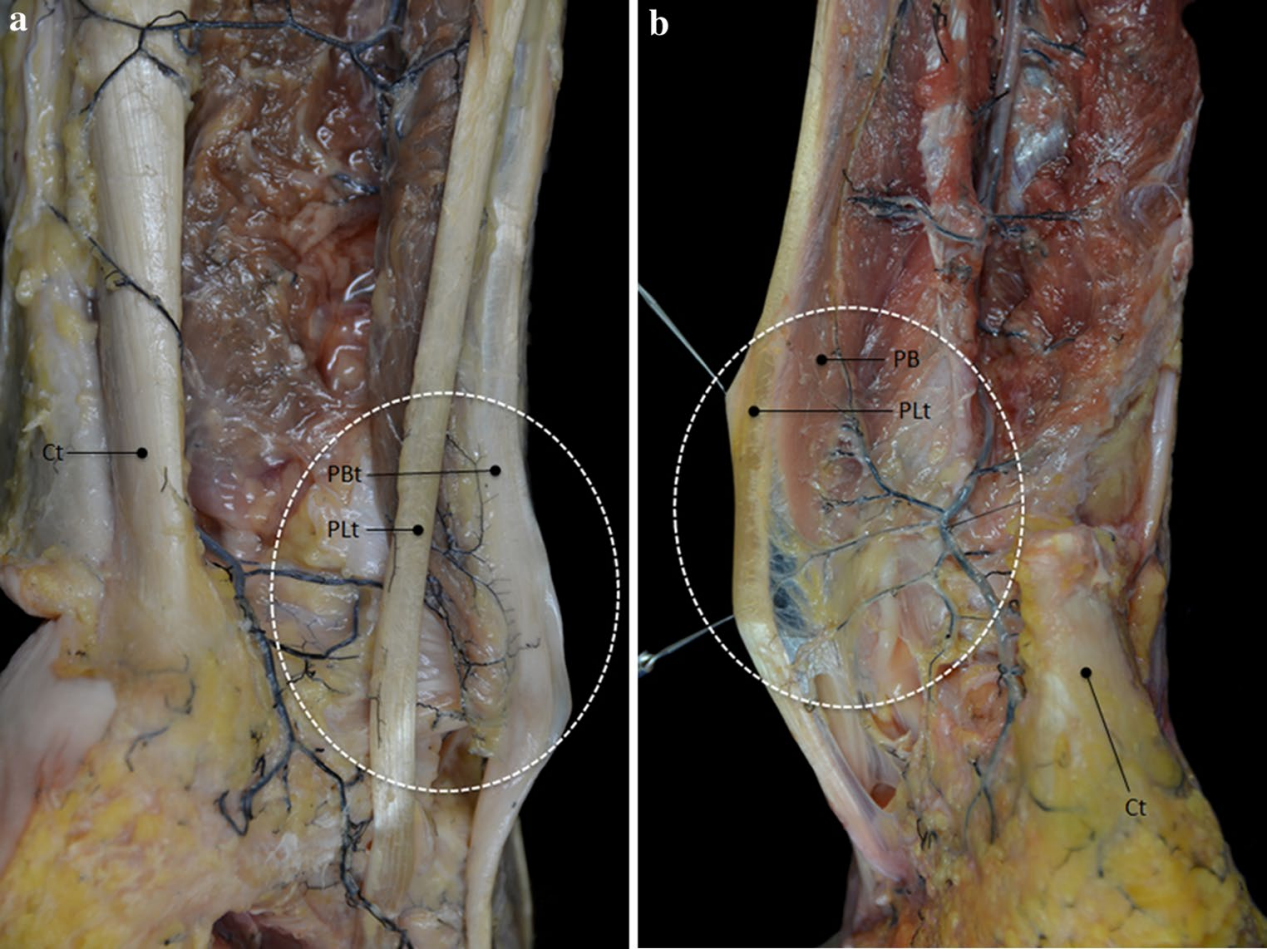
**a** In 8 cases, vessels from the peroneal artery form an arcuate pattern on the vincula before entering the peroneal tendons. *Ct* calcaneal tendon, *PBt* peroneus brevis tendon, *PLt* peroneus longus tendon. **b** In 2 cases, branches of the peroneal artery form a weblike structure on the vincula before entering the peroneal tendons. *PB* peroneus brevis muscle, *PLt* peroneus brevis tendon, *Ct* calcaneal tendon

A common vincula attached to the posterior side of the tendons connected both tendons and played an important role in their blood supply. Vessels reaching the vincula trough the peroneal muscles could be distinguished into two different vascularization patterns: an arcuate pattern (Fig. 5a) or a weblike network (Fig. 5b).

## Patterns of arterial supply

**Fig. 6:**
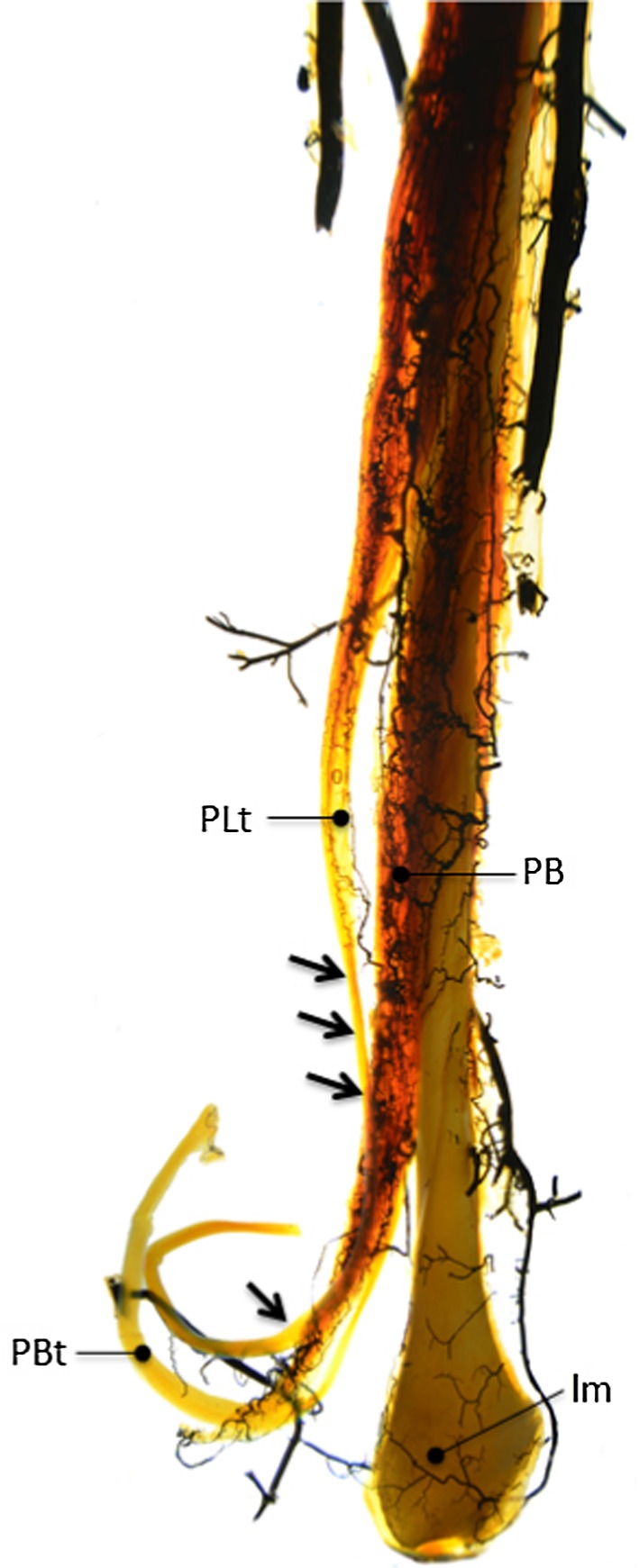
In 8 cases, the Spälteholz technique visualized well vascularization of the PB tendon along the whole course of the tendon. Poor vascularized zones within the PL are found in the retromalleolar groove and 2–3 cm proximal to the retromalleolar groove (*arrows*). *PLt* peroneus longus tendon, *PB* peroneus brevis muscle, *PBt* peroneus brevis tendon, *lm* lateral malleolus

In 8 out of 10 specimens, branches of the peroneal artery penetrated the muscles of both the PB and the PL tendon. Vessels ran from proximal to distal, ending in the proximal part of the peroneal tendons. Some vessels entered the vincula, forming a dense arcuate pattern on the surface of the PB tendon and giving rise to small collateral vessels penetrating the PL tendon through the connective tissue that joins both tendons (Fig. 5a, b). Poor vascularized zones within the PL tendon were found in the retromalleolar groove and 2.0–3.0 cm proximal to the retromalleolar groove (Fig. 6). The PB tendon was well vascularized over the whole course of the tendon, without appearance of avascular zones. The vascular density within the PB tendon was clearly higher than in the PL tendon.

**Fig. 7:**
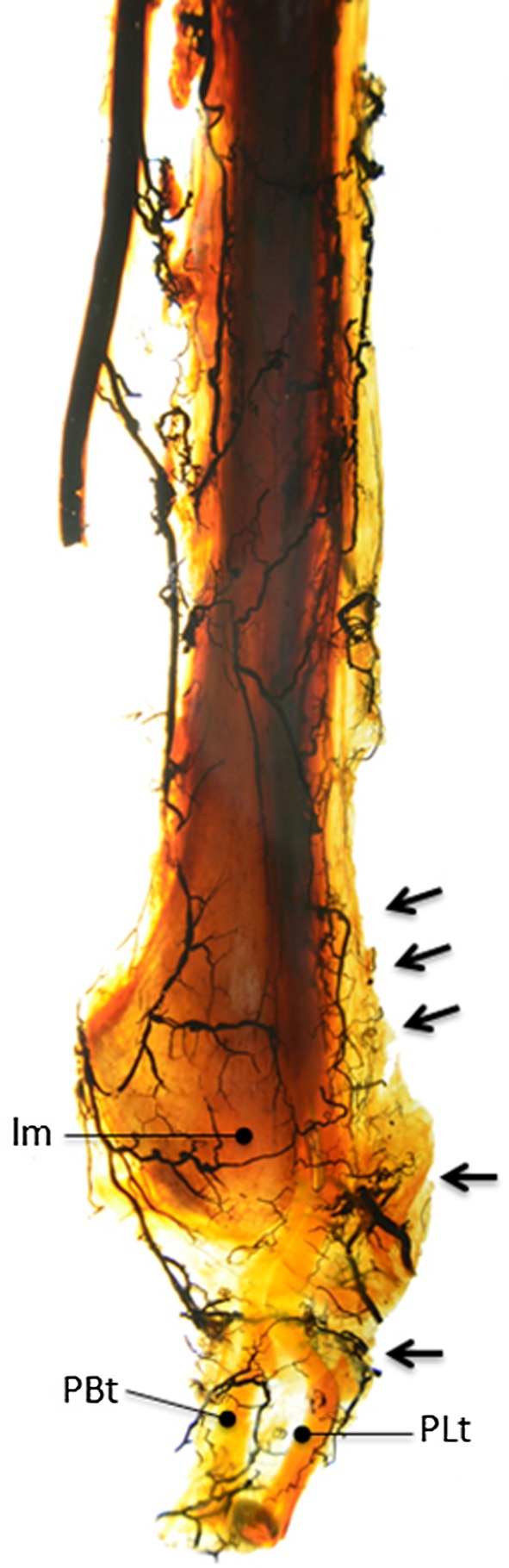
In 2 cases, the Spälteholz technique showed well vascularization of both the PB and the PL along the whole course of the tendons (*arrows*). *lm* lateral malleolus, *PBt* peroneus brevis tendon, *PLt* peroneus longus tendon

In 2 out of 10 specimens, both muscles were directly vascularized by branches from the peroneal artery. Vessels entered the vincula, forming a weblike network over the whole length of both tendons. In these specimens, no avascular zones were found in both the PB tendon and the PL tendon (Fig. 7).

## Discussion

The results of the current study suggest that both peroneal tendons are well vascularized, with a clearly higher vascular density in the PB tendon relative to the PL tendon. The tendons are mainly vascularized by the peroneal artery, via small vessels running through a common vincula. After entering the vincula, two vascularization patterns could be distinguished: either (1) small vessels formed a dense arch on the surface of the PB tendon, giving rise to small collateral vessels penetrating the PL tendon, or (2) branches entered the vincula forming a weblike network and then perforated both tendons. No avascular zones could be distinguished in the PB tendon. In the PL tendon, poor vascularized zones were only found when an arcuate structure could be distinguished.

To gain knowledge on the pathophysiology of peroneal tendon pathologies and aid surgical approaches, understanding of the vascularization of the peroneal tendons has been looked over previously [19, 26]. The results of the current study regarding the major blood supply of the tendons are in line with previous findings. Both peroneal tendons are mainly vascularized by branches of the peroneal artery. Results of the current study suggest that not the medial tarsal artery but the lateral tarsal artery in some cases anastomoses with the perforating branch of the peroneal artery, contributing to the vascularization of the peroneal tendons at the dorsolateral region of the tarsus.

As proposed by Scholten and van Dijk [24], the current study found branches of the peroneal artery reaching the tendons trough a common vincula attached to the posterior side of both tendons. Vincula are described as synovial tissue, connecting the tendon to their tendon sheath [13]. The vincula of the peroneal tendons is attached to the dorsolateral aspect of the fibula and continues until the distal insertion of the tendons [31]. In vascularization of flexor tendons of the hand and the anterior tibial tendon, vincula have been proven to be of great importance in blood supply [10, 12]. Facilitating the vascularization of the peroneal tendons, surgeons should be aware of the location of the vincula and ensure that it remains intact.

In 1992, Sobel et al. [26] found vascular supply by the peroneal artery over the whole course of both tendons by injecting Indian ink into the arteries. No evidence was found for avascular zones. Eight years later, Petersen et al. [19] reported three critical avascular zones within the tendons. They stated that the method used by Sobel et al. was not accurate. Ink may leak into the intervessel area due to high pressure or damaged vessels, creating false-positive results. On the other hand, microembolism, low pressure and hardening of the injection medium before it reaches the terminal arteries may cause inadequate filling of the vessels leading to false-negative results [19]. Petersen injected Indian ink combined with gelation in the arteries to visualize the vascularization. Therefore, discrepancy between the two studies could be explained not only by false positives in the results from Sobel et al., but also by false negatives in the results from Petersen et al.

In an anatomical study of Edwards [8], the vascular network within tendons in general was determent as longitudinal vessels with transversal connections running trough the entire length of the tendon. Such distribution pattern was also found in the current study. Vascular injection showed a homogeneous vascular distribution and a dense vascular network in the PB tendon, corresponding to the study from Sobel et al. [26]. In the PL tendon, poor vascularized zones were found around the lateral malleolus in the cases were small vessels first passed the PB tendon and the vincula before reaching the PL tendon. This is more in line with the study from Petersen et al. [19]. Discrepancy between the results of the current studies and earlier studies could be explained by the false-negative and false-positive effects of injection techniques. Difference in accuracy of the different methods may also explain the difference.

Peroneus brevis tendinopathy mostly occur around the fibula [21, 25]. Petersen et al. [19] found avascular zones corresponding with the most common sites of tendinopathy and concluded that poor blood supply is related to PB tendon tears. However, the literature shows controversy on the relation between blood supply and tendon ruptures [23]. In Achilles tendon ruptures, for example, several authors doubt the relationship between blood supply and frequency of ruptures [1, 2, 17, 18, 23]. In the current study, no avascular zones were found in the PB tendon. Therefore, the relationship between vascularization of the peroneal tendons and the location of tears is questioned. A different proposed mechanism of tendon injury is structural disturbance of the tendon due to stress [23]. With the PB tendon squeezed in between the PL tendon and the bony pulley at the level of the retromalleolar groove, the zone where most PB tendon tears occur, frequency of tears in different zones of the tendon may be explained by high stress and pressure in the groove [21, 30].

The literature shows that increased vascularization is often found in chronic tendinopathies. Chronic tendon pathologies seem to be a highly active process when it comes to neovascularization of the tissue. Tenosynovitis, the precursor of chronic PB tendon tears, is associated with neovascularization. It is unknown why healing of tendinopathies and their hyper vascular state tends to fail. However, it is known that invasion and proliferation of new blood vessels may contribute to pain and chronicity of a tendon disorder [9].

The limitations of this study should be taken into account. First, the use of cadavers carries possible inherent bias. Findings obtained from cadavers may differ from the in vivo situation. Also, freezing and thawing can damage the tendons and therefore influence results [5]. However, to determine the pattern of blood supply to and within a tendon, a cadaveric study seems to be the most accurate study design.

Another limitation is the low number of specimen. Anatomical variations may have influenced our results, and therefore, a higher number of specimens would lower the possible change incidental findings.

A third limitation is the lack of immunohistochemical demonstration of laminin within the tendon, which would have put more weight to our results.

## Conclusion

The peroneal tendons are well vascularized by distal branches of the peroneal artery, running through a common vincula and no avascular zones could be distinguished in the PB tendon. Vessels from either the fibular artery or the perforating branch of the peroneal artery contribute to vascularization of the tendons on the dorsolateral region of the tarsus. To keep the tendons well vascularized and therefore improve tendon healing, surgeons should be careful leaving the vincula intact during surgical procedures.
